# An Ishmaelite

**Published:** 1888-07-07

**Authors:** Leslie Keith

**Affiliations:** Author of "The Chilcotes," "Uncle Bob's Niece," "East and West," etc.


### An Ishmaelite.

By Leslie Keith,

Author of "The Chilcotes," "Uncle Bob's Niece," "East and West," etc.

(Continued from p. 218.)

### Chapter XIII.

The yoke to which Arthur had yielded so lightly was beginning now to fret the wearer, and to gall him in tender places. Arthur was conscious of the strain, and he was ashamed of his rebellion, and repented only to transgress again.

When he was cold to Ella, or inattentive to her placid outpourings, he did penance by lying awake at nights, or sitting up to smoke a cigar over the rehearsal of all her good qualities. Those virtues of hers, how often he trotted them out, and told himself how little worthy of her he was—her amiability, her good sense (that family inheritance), her unexacting disposition. "She doesn't want much of me," thought poor Arthur. "Why can't I give her what she wants? She is so goodnatured "—then the catalogue would begin again. Was it because the prize was growing of less value in his eyes that he so anxiously summed up all its merits?

Ella had some reason to be dissatisfied with her lover; she might have argued with perfect truth that she was just as she always was, of the same lukewarm, equable temperature. She had not been more ardent when he had declared his love for her, and he had mistaken the beating of his own heart for hers—that was all. The latest cause of disagreement between the pair was Arthur's refusal to go down to Norfolk. The moment for Ella's annual visit to her home arrived with August, and Ella was a person who loved precedent and order and a fixed habit of life.

"You ought to come with me, Arthur," she said. "You haven't been in my home since we were engaged."

"It is impossible for me to go this summer."

"Why impossible?" she asked with mild persistency. "You can do as you like at the office, you are a privileged person, and you can usually find time to go to Scotland for the shootings."

"I don't intend to go to Scotland this year," said Arthur inwardly groaning, but trying to summon patience. "I've a reason for remaining in town."

"Nobody stays in town in August."

"A few millions usually do, Ella; the town isn't emptied, my dear, because you go to Norfolk." Arthur was ashamed of the sneer the moment he had uttered it. He went up and took her hand and looked at her very kindly. "Ella, dear, don't let us quarrel over this," he said. "I can't go, and there's an end of it. You will enjoy yourself very well without me."

"But, Arthur," her desire to prevail overcame her tact, "I don't think it is very respectful to papa and mamma. They have invited you, and they expect you. I really think it is your duty to go."

His face hardened a little under her words, but he still held her hand and tried to speak kindly.

"Surely I am the best judge of what is my duty," he said; "and, Ella, I think you must guess, you must know what it is that keeps me in town—who it is. You have your parents and your brothers and sisters and all your old friends to go to, but there is someone here who has no friend in London but me, and whom I cannot forsake."

He spoke with reined-in feeling, but her immobile face gathered no emotion from his. She looked at him with the same steady light in her calm blue eyes, and her only sign of a lessening temperature lay in the gentle withdrawal of her hand from his.

"Of course I understand you, Arthur," she said. "I have not failed to notice that you leave me every evening now, and I could scarcely help guessing to whom it is you go. I have not complained, though. Cousin Joseph has remarked your absence too, and it is not very pleasant to have other people taking notice of your neglect."

"Neglect!" cried Arthur, hotly. "If I neglect you, it is because you are so inf——" He pulled himself up, and bit his lip. "You make it so awfully hard for a fellow; you have no sympathy."

"You couldn't expect me to have any sympathy with you on such a subject," she said, with a primness and propriety that embittered his mood. "I scarcely know what you wish of me. Do you propose that I should go with you—to the slums, or wherever it is you go—to visit your cousin?"

"If I were ever foolish enough to dream of such a thing, I am wiser now," he said, bitterly. "I might have asked another woman, but not you, Ella."

"You could never have asked a lady," she said, with mild emphasis. "Even if I wished it, papa and mamma would not like it; and really, Arthur, when you so curtly refuse so reasonable a request as mine, I fail to see how you can be angry with me for declining to yield to you when you are unreasonable."

"Perhaps I am unreasonable," said Arthur sadly, ashamed of this constant bickering, and sore at being misunderstood. "I will go with you for a couple of days, if that will satisfy you, Ella, and appease the proprieties"—he could not resist the addition—"and I will not ask you to go with me, my dear, for I think you are quite right; the slums, as you call them, are no place for you."

"I am glad you agree with me at last," said Ella, with perfect self-complacency and equanimity; "and though two days isn't much of a visit, I don't wish to be exacting. It is better than nothing, and perhaps you will see your way to remaining a little longer. It really is the right thing to do."

"By all means let us do the right thing," said Arthur. He kissed her, penitent over his own share in the estrangement, but with an unhealed ache at his heart. Ella had mastered him; at least, she had made him surrender for peace's sake; but if every difference of opinion between them was to be fought out with an equal unyieldingness, surely their future held but scant hope of comfort.

To Arthur it seemed to bristle with hazards. Married people can be tolerably reckless of each other's feelings, it is true, and they survive an exchange of plain speech that would certainly estrange them if they were bound by any less close tie, but where is the young man as yet only a lover, who can calmly contemplate a married life of ceaseless bickering, and be content to think it inevitable? When Arthur won his Ella, didn't he think himself the happiest fellow in London? the most to be envied? Didn't he long for the day when he could call her his own? How was it that he could scarcely think of that day now without a sinking of heart; could not construct any future that was not full of small jars and ugly frets; alienated looks across the table at breakfast, polite skirmishes at lunch, a cold silence at dinner? He did not relish the look of the drama, nor his own sordid part in it.

"It is really all your fault," Ella often used to say, when he came to her penitent and remorseful after one of his fitful moods; and no doubt she was right. The worst of the Ella-like people, with their abominable coolness, inflexibility, and self-possession, is that they are so often right.

When Ella had been a little more aggravating than usual, Arthur had a refuge to which he could escape. It was not in the slums, as we know, though it was still far enough removed from the beaten tracks of his life, too widely removed perhaps to be quite a safe shelter for a man of his impulsive fibre. Fred, who was beginning to wake up to a new consciousness of many things, had a glimmering comprehension of this too. Arthur's visits to the library were mostly made in the evening, when the solemn family dinner at Portland Place, of which Ella would permit no curtailment, was over. By that hour of the summer night custom had almost ceased to flow to the library, though the neighbouring restaurant was still busy, and the librarians were left to reckon up the day's outgoings, and rearrange the thinned ranks on the shelves. When Arthur came, Barbara generally made some excuse to slip away and leave the cousins alone.

On one of these occasions Fred made his remonstrance.

"Arthur," he said, "here is August and you are still in London."

"Is there any law against my being here?" said Arthur with a smile. "It seems to me there are still a good lot of folk about."

"You ought to be out of town."

"Well, I am going out of town. I am going to Norfolk on Saturday."

"I am very glad to hear it."

"And I shall return to London on Monday. I hope you are glad to hear that too."

Fred shook his head.

"I wish you were coming with me," Arthur suddenly burst out. "Not to Norfolk, I couldn't wish my worst enemy a heavier punishment than to spend a week in the rectory with cousin Fanny—not to Norfolk; not anywhere in this wornout old England, but to a new country, where Mrs. Grundy hasn't penetrated, where there are no cousin Fanny's."

Fred smiled, but he looked very grave the next moment.

"You don't like the Norfolk business," he said, "but when you've paid your debt to family duty, you'll go north and smell the peat-reek, and see the heather, and have a shot at the birds. You will go and not come back for a month or two."

"Do you mean that you want to get rid of me, Fred?"

"Yes, I mean that," said Fred steadily; "I can do without you, and the less you see of me, the better it will be for you. What can you gain by coming here, my boy? Nothing but the disapproval of your best friends: stick to them, Arthur, and believe them when they tell you this is no place for you."

"Have you joined the multitude too?" said Arthur with some scorn. "Have you got hold of that cant about keeping one's station, and seeking your acquaintances only among people who can give you a leg up? I thought all that talk was woman's nonsense."

"My station! What station have I? If I had my deserts, do you think I should be here? If my story came to be known, how long do you think it would be possible for me to remain among decent, self-respecting people? But you have done nothing to make you humbly grateful for such a shelter as this, you have the world to choose from."

"And if I choose this for my world? Am I a girl in the schoolroom that I should have my associates chosen for me, my friendships passed under the review of papa and mama? Fred, it is useless. If you will not go with me to a new world, you must accept me as part of your world here. My lot isn't so particularly jolly that it requires any tremendous amount of selfdenial on my part to quit it for a little."

"You ought not to quit it; you have ties there and duties."

"Don't turn moralist; don't I know my duty: am I not going to Norfolk?"

But if Arthur knew his duty, why, when he had seen Fred, did he not go back to it? It was Fred he came to see—yes, and truly he loved this erring hero of his with a love surpassing that he felt for woman: it was still the strongest and most compelling affection of his life, and it was for Fred's sake in the beginning that he took an interest in the little household of which he formed part.

Arthur liked to go up to the low room, with its queer, archaic furniture, its gropings after a better standard than the neighbourhood offered, its odd inhabitants, with Delia for the central attraction. How pretty the child was, and how modest too. He liked to watch her as he would have watched some pet plaything. He took note of all her graceful turns and motions—her shy, startled glances—her sudden blushes. The young man saw these things with that reverence and chivalry for women that even Ella had been able to waken in him. Not for the world would he have startled her by so much as a look that seemed too bold.

Miss Betsy amused him and interested him in another way; and so did the simple host. It seemed to him as if they had preserved an individuality and originality that had been crushed out of the set he was used to. "If I could write about them just as they are, what a book I might make," he thought.

The daughters had fewer differences, except that they were better informed than many girls he had met, and more modesty over it. Ella, whose admirable qualities never needed so much rehearsal on his part as when he paid a visit to the home above the shop, probably harboured less love in her neat and tidy head than Barbara had in her little finger. But she was far handsomer than Miss Barbara. It gave him a sort of pleasure to discover this.

Once, when he was alone for an instant with the younger girl, he asked her very respectfully where she got her pretty shepherdess's name.

"It was the name of a lady who was my mother's mistress," Delia said simply. "Mother was housekeeper there for years."

"One of the Grahams? To be sure, I remember a younger Delia Graham. They are relations of mine. I think it must have been my grand-aunt you were named for."

"I hope you don't mind my having her name?" said Delia, with one of her vivid blushes.

"It suits you a great deal better than it suited her," he said. Indeed, it suited her to perfection.

Delia!—he had laughed at the name once, but he found it very pleasant to the ear now. And it was odd that this pretty child should be named for his grand-aunt. He found it quite a striking coincidence.

*(To be continued.)*

